# Anatomic versus reverse total shoulder replacement for patients with osteoarthritis and intact rotator cuff: the RAPSODI-UK randomised controlled trial protocol

**DOI:** 10.1136/bmjopen-2025-106740

**Published:** 2025-12-12

**Authors:** Hannah L Rodrick, Joseph Dias, Adam C Watts, Michael J Walton, Stephen Brealey, Richard Page, Nadine E Foster, Katy Boland, Lindsay J Cunningham, Caroline Fairhurst, John Geoghegan, William Greenwood, Catherine Hewitt, Cliona Kirwan, Heather Leggett, Catriona McDaid, Matthew Parkes, Steve Parrott, Rachael Powell, Jonathan G Quicke, Gareth Roberts, Fiona Rose, Harvinder Pal Singh, Helen Spickett, Chris J Sutton, Fraser Wiggins, Qi Wu, Ian Trail

**Affiliations:** 1 Department of Health Sciences, York Trials Unit, University of York, York, UK; 2 Department of Trauma and Orthopaedics, University Hospitals of Leicester NHS Trust, Leicester, UK; 3 Musculoskeletal Research Group, Edge Hill University, Ormskirk, UK; 4 Upper Limb Unit, Wrightington Wigan and Leigh Teaching Hospitals NHS Foundation Trust, Wigan, UK; 5 Department of Orthopaedic Surgery, Deakin University - Geelong Waterfront Campus, Geelong, Victoria, Australia; 6 STARS Education and Research Alliance, The University of Queensland and Metro North Health, Brisbane, Queensland, Australia; 7 The School of Health and Rehabilitation Sciences, Faculty of Health, Medicine and Behavioural Sciences, The University of Queensland, Brisbane, Queensland, Australia; 8 Department of Research & Development, Wrightington Wigan and Leigh Teaching Hospitals NHS Foundation Trust, Wigan, UK; 9 Nottingham Shoulder and Elbow Unit, Nottingham University Hospitals NHS Trust, Nottingham, UK; 10 Patient and Public Involvement (PPI), Wrightington Wigan and Leigh Teaching Hospitals NHS Foundation Trust, Wigan, UK; 11 Division of Cancer Sciences, The University of Manchester, Manchester, UK; 12 Centre for Biostatistics, University of Manchester, Manchester, UK; 13 Division of Psychology and Mental Health, School of Health Sciences, The University of Manchester, Manchester, UK; 14 Applied Health Research Unit, University of Lancashire, Preston, UK

**Keywords:** Orthopedics, Pragmatic Clinical Trial, Randomized Controlled Trial, Shoulder, Orthopaedic & trauma surgery

## Abstract

**Introduction:**

Shoulder osteoarthritis most commonly affects older adults, causing pain, reduced function and quality of life. Total shoulder replacements (TSRs) are indicated once other non-surgical options no longer provide adequate pain relief. Two main types of TSRs are widely used: anatomic TSR (aTSR) and reverse TSR (rTSR). It is not clear whether one TSR type provides better short- or long-term outcomes for patients, and which, if either, is more cost-effective for the National Health Service (NHS).

**Methods and analysis:**

RAPSODI-UK is a multi-centre, pragmatic, two-parallel arm, superiority randomised controlled trial comparing the clinical- and cost-effectiveness of aTSR versus rTSR for adults aged 60+ with a primary diagnosis of osteoarthritis, an intact rotator cuff and bone stock suitable for TSR. Participants in both arms of the trial will receive usual post-operative rehabilitation. We aim to recruit 430 participants from approximately 28 NHS sites across the UK. The primary outcome is the Shoulder Pain and Disability Index (SPADI) at 2 years post-randomisation. Outcomes will be collected at 3, 6, 12, 18 and 24 months after randomisation. Secondary outcomes include the pain and function subscales of the SPADI, the Oxford Shoulder Score, health-related quality of life (EQ-5D-5L), complications, range of movement and strength, revisions and mortality. The between-group difference in the primary outcome will be derived from a constrained longitudinal data analysis model. We will also undertake a full health economic evaluation and conduct qualitative interviews to explore perceptions of acceptability of the two types of TSR and experiences of recovery with a sample of participants.

**Ethics and dissemination:**

Ethics committee approval for this trial was obtained (London - Queen Square Research Ethics Committee, Rec Reference 22/LO/0617) on 4 October 2022. The results of the main trial will be submitted for publication in a peer-reviewed journal and using other professional and media outlets.

**Trial registration number:**

ISRCTN12216466.

**STRENGTHS AND LIMITATIONS OF THIS STUDY**

- RAPSODI-UK is a multi-centre, pragmatic, two-arm, parallel, superiority randomised controlled trial with a parallel sister trial being conducted in Australia.
- This study includes a full health economic evaluation and a nested qualitative interview study of participants’ experiences.
- There will be a 2-year follow-up and beyond using National Joint Registry (NJR) data linkage for longer term follow-up.
- There is the potential cross-over of randomised participants from anatomic to reverse Total Shoulder Replacement due to a lack of rotator cuff integrity when assessed intra-operatively.

## Introduction

### Background and rationale

 Osteoarthritis (OA) of the ‘ball’ (humerus) and ‘socket’ (glenoid) joint of the shoulder is common with advancing age. Resulting shoulder pain and disability significantly impact patients’ work, social and domestic activities, contributing to a considerable burden on healthcare systems.^1^ A total shoulder joint replacement (TSR) may be appropriate when other non-surgical options no longer provide adequate pain relief. In 2023, 8821 primary TSRs were performed across England, Wales and Northern Ireland, with 55% of these procedures attributed to shoulder OA.^2^

There are two primary types of TSR: anatomic (aTSR) and reverse (rTSR). Anatomic TSR aims to preserve the natural anatomy of the shoulder joint, making it suitable for patients with an intact rotator cuff and normal functioning shoulder muscles that enable lifting the arm above shoulder height. In contrast, rTSR reverses the orientation of the joint components—placing the ‘ball’ on the glenoid and the ‘socket’ on the humerus—and relies on the deltoid muscle for arm elevation. This approach is often indicated for patients with a deficient or dysfunctional rotator cuff, as it compensates for rotator cuff deficiency. Despite its clinical success, aTSR is associated with subsequent rotator cuff deficiency,^3^ which is one of the leading causes of revision surgery. As a result, more patients with an intact rotator cuff are being treated with rTSR, even in the absence of clear evidence demonstrating its superiority over aTSR. While rTSR is increasingly being performed in clinical practice,^2 4^ there are insufficient data regarding its cost-effectiveness compared with aTSR, particularly in terms of hospital costs. Data from the American healthcare system suggests that there may be higher hospital costs associated with rTSR, making it essential to better understand the relative benefits and economic implications of the two procedures.^5 6^

The current evidence base is limited, with a Cochrane review in 2020 reporting a lack of high-quality randomised controlled trials (RCTs) comparing aTSR and rTSR in patients with shoulder OA and an intact rotator cuff.^7^ Data from the National Joint Registry (NJR)^2^ indicate that the risk of revision of aTSR for cuff failure is 0.42 per 100 prosthesis years compared with rTSR with a rate of 0.02 per 100 prosthesis years. More recently, a Korean group undertook a systematic review and meta-analysis of six retrospective studies of 447 patients with intact rotator cuffs for primary shoulder OA receiving either aTSR or rTSR.^8^ They reported that range of movement (ROM) was better (specifically external rotation, although there was tentative evidence for better ROM in all tested directions) for aTSR compared with rTSR. While function scores were not different, glenoid loosening (the failure of the glenoid component of the prosthesis to remain securely attached to the bone) was more common with aTSR, whereas scapular notching (erosion of the inferior scapular neck due to repetitive mechanical abutment of the humeral component during adduction) only occurred with rTSR. Other surgical risks were similar for the two types of implant, and overall, there was no difference in revision rate. However, the included studies were all retrospective, and many suffered from methodological limitations, including short follow-up and small sample sizes. Therefore, considerable uncertainty remains as to whether rTSR leads to better and more enduring outcomes compared with aTSR in this patient group.

In 2015, the James Lind Alliance priority setting partnership for shoulder surgery identified a comparison of different types of shoulder replacement for shoulder arthritis as a research priority.^9^ A 2020 review commissioned by the National Institute for Health and Care Excellence (NICE) found that no RCTs had compared the clinical or cost-effectiveness of aTSR versus rTSR in patients with shoulder OA and an intact rotator cuff. As a result, NICE recommended an RCT to address this gap in the evidence base.^10^ The RAPSODI-UK trial was designed in response to a commissioned call from the National Institute for Health Research Health Technology Assessment programme for a pragmatic trial to provide the high-quality evidence that has been previously lacking in this area.

The RAPSODI-UK trial will also include a nested qualitative study to explore patient perceptions of acceptability of the interventions, their experiences of recovery and patients’ goals at two postoperative time points (approximately 2 and 12 months after surgery). It is expected that this nested study will provide valuable contextual information to further understand the RCT results.

### Aims and objectives

The overall aim of the RAPSODI-UK RCT is to provide robust evidence that can guide clinical decision-making and inform healthcare policy regarding TSR for shoulder OA in older adults. The specific objectives are listed in box 1.

Box 1 **Objectives of the RAPSODI-UK trial**

1. To determine whether reverse total shoulder replacement (rTSR) is superior to anatomic total shoulder replacement (aTSR) for the treatment of painful osteoarthritis (OA) of the shoulder joint with an intact rotator cuff and suitable bone stock in patients aged 60 years and over as measured by patient-reported pain and function using the Shoulder Pain and Disability Index (SPADI) at 24 months.
2. To obtain estimates about recruitment rate and assumptions regarding cuff integrity and whether this leads to crossovers from aTSR to rTSR via an 8-month internal pilot.
3. To compare the cost-effectiveness of the two treatment options to determine the most efficient provision of future care and to describe the resource impact on the National Health Service (NHS) for both treatments.
4. To explore patients’ perceptions of acceptability of aTSR and rTSR, patients’ goals, and their experiences of recovery, within and across trial groups.

## Methods

This protocol is reported in line with the Standard Protocol Items: Recommendations for Interventions Trials (SPIRIT)^11^ and the Consolidated Standard of Reporting Trials (CONSORT) guidelines.^12^

### Trial design

**Figure 1:**
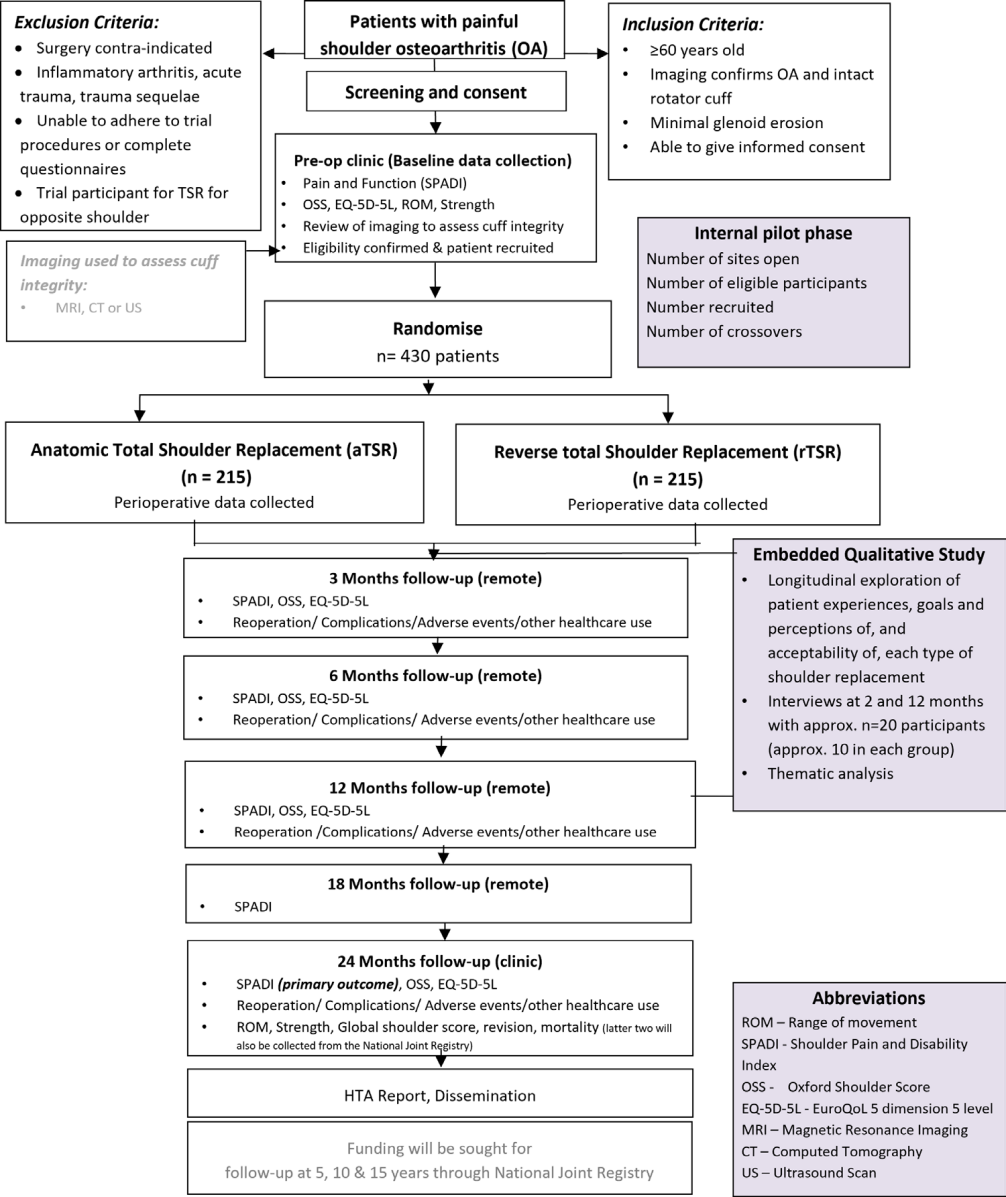
Overview of trial design and flow of participants through the trial.

RAPSODI-UK is a multi-centre, pragmatic, two-parallel arm, patient and assessor blinded, superiority RCT with internal pilot (see online supplemental file for further details relating to the internal pilot and progression criteria). The participant flowchart can be seen in figure 1.

An accompanying health economic evaluation, and a nested qualitative study with a subset of trial participants will be included.

### Setting

Participants will be recruited from orthopaedic departments of NHS Hospitals in England, Wales and Northern Ireland that routinely treat shoulder OA with both aTSR and rTSR. We will collaborate with approximately 28 high and medium volume hospital sites and will prioritise those that have performed at least 130 TSRs in the 3 years preceding 2019. Linkage to NJR data will be used to compare mortality and revision rates at follow-up points beyond 2 years. Scottish sites will not be included given the need to link data to the NJR dataset.

### Eligibility criteria

Eligibility criteria are presented in box 2.

Box 2 **Participant eligibility criteria for the RAPSODI-UK trial**

**Inclusion criteria**

- Aged 60 years and over.
- Diagnosis of painful osteoarthritis (OA) of the glenohumeral joint, confirmed by routine radiographs, that has not been controlled by previous interventions.
- An intact rotator cuff determined by pre-operative advanced imaging (ultrasound, MRI or CT).
- Minimal glenoid erosion determined by pre-operative CT or other imaging in whom an off-the-shelf replacement is appropriate.
- Able to give informed consent.

**Exclusion criteria**

- Shoulder replacement surgery contra-indicated.
- A diagnosis of inflammatory arthritis, acute trauma or trauma sequelae.
- Evidence that the patient would be unable to adhere to trial procedures or complete questionnaires.
- Trial participant for TSR for the opposite shoulder.

### Recruitment

Potential participants will be screened and identified from the waiting lists for TSR prior to the pre-operative clinic, or from those patients attending an outpatient clinic. The research team will work closely with the delegated hospital staff (eg, surgeons, nurses, physiotherapists) at each participating site to optimise the local screening and recruitment processes.

Radiographs (typically anteroposterior and axial views) to confirm OA of the shoulder joint will have been taken as part of routine care. More advanced routine imaging with CT, MRI or ultrasound carried out within 6 months of surgery, where possible, will be used to assess the integrity of the rotator cuff. Earlier scans can be used if scans within 6 months are not available as part of local routine care. A routine CT scan will be used to determine the extent of glenoid erosion.

Delegated hospital staff will screen and approach eligible patients to take part in the trial. For new patients, this will be at their outpatient appointment clinic. For waiting list patients, this may require bringing the patient in for an outpatient appointment or calling the patient and/or posting to them the RAPSODI-UK trial patient information leaflet. Posters about the trial will also be displayed in clinics. Informed consent will be confirmed and baseline data collected when the patient has been confirmed fit for surgery at the pre-operative assessment clinic or consent to surgery clinic by a suitably qualified and delegated member of the research team. Where feasible, this will be done within 6 weeks from the planned surgery.

Consent can occur face-to-face during a clinic appointment or alternatively, can be carried out remotely via phone or video call. Remote consent will be carried out by a GCP-trained staff member and witness. On the remote consent call, the patient will have the option to verbally consent and be posted the baseline forms to complete and return to York Trials Unit via prepaid post. When the patient next comes into the hospital, they will confirm their willingness to participate and physically sign the Verbal Consent Form.

#### Sequence generation and allocation concealment and implementation

Allocation will be 1:1, using random permuted blocks of random block size, stratified by age (60-69; 70+) as a surrogate of deteriorating shoulder rotator cuff function. The allocation schedule will be generated by a trial statistician, otherwise not involved in the recruitment or randomisation of participants. It will be implemented using a secure web-based randomisation service managed by York Trials Unit, ensuring treatment concealment and unbiased allocation. The research team at the site will access the online service to perform the randomisation ideally 2 weeks before surgery but no earlier than the pre-operative clinic to confirm the patient is fit for surgery.

#### Blinding

Participants will be blinded to treatment group allocation and will not be told which TSR type they have received. This is feasible given that the scars of the two TSR types appear the same. Participants will be provided with a card to remind them about their blinding and to remind healthcare professionals at appointments about being blinded. Sites will also be provided with a generic leaflet about helping participants recover from their operation which is not specific to TSR type. To help prevent unblinding of participants from occurring, we will ask sites to list participants as a ‘RAPSODI-UK Total Shoulder Replacement’. If a participant inadvertently becomes unblinded to their allocation, this will be recorded. Surgeons performing the surgery cannot be blinded to allocation. Outcome assessors who will undertake the shoulder ROM and strength of shoulder measurements will be blinded to the replacement type the participant received. Blinded outcome assessors will be asked not to access radiographic records of the participant as these do not form part of the outcome assessment for the patient. The primary outcome is a patient-reported measure (SPADI), helping mitigate surgeon influence. Participants will be informed which type of surgery they had after primary outcome data are collected at 24 months. We will remind site staff on hospital Case Report Forms not to unblind patients and to record if it does happen. Participants will only be unblinded earlier than 24 months if there is deemed to be a clinical need by their surgeon.

Trial participants will also be asked at 24 months whether they have been informed of the type of replacement they have had during the trial, and which type they thought they had received. Participants may, however, request to withdraw from being blinded and be provided with their allocation: any such participants will continue to be followed up, but information on their unblinding will be recorded.

### Trial training

Trial coordinators will meet virtually with delegated NHS research teams via video call at each proposed RAPSODI site in order to discuss the study and provide training of specific processes. Sites will also be provided with guidance on how to collect the ROM and Strength measures using the equipment provided by the Sponsor. Trial coordinators will continue to liaise with sites during recruitment to ensure adherence to the protocol, completion of data collection and training of new staff members, as applicable.

#### Interventions

For both interventions, we will only include commercially available, off-the-shelf implants. We define ‘off the shelf’ as not designed or bespoke but taken from existing stock or supplies. Some hospitals will use fluoroscopic imaging during surgery, as part of their standard practice.

To reflect the pragmatic design of this trial, a required level of experience of the operating surgeon will not be defined, although all surgeons performing TSR for patients within the trial will be required to be familiar with the techniques and equipment that they are using. Data will be collected on the grade and experience of the primary and secondary surgeons through the NJR minimum data set.

**Figure 2:**
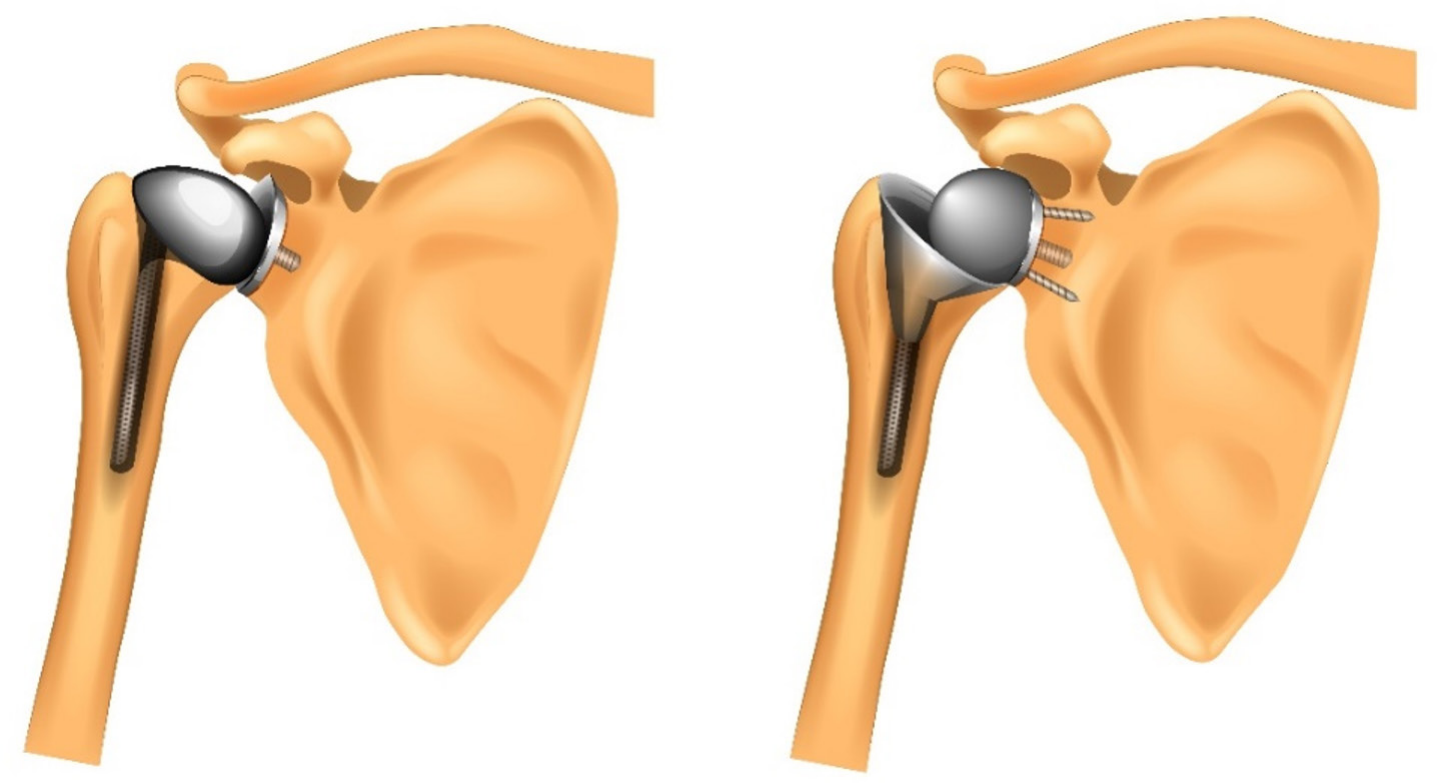
Diagram of a total shoulder (anatomic) replacement (left) and a reverse shoulder replacement (right).

The treatment of the rotator cuff during the surgical approach and repair on completion of the surgery will be recorded but will be left to the discretion of the operating surgeon. The surgical approach may include a deltopectoral or deltoid splitting approach and will depend on local practice at recruiting sites. This will not be mandated in the protocol in keeping with the pragmatic design but will be recorded. There may be some patients who are initially randomised to aTSR whose rotator cuff is deemed unsuitable at surgery and thus receive rTSR instead. These patients will be followed up under intention-to-treat and the incidence of these crossovers will be monitored throughout. Figure 2 illustrates the two types of TSR that are described below.

##### Anatomical total shoulder replacement

The aTSR is a conventional TSR which mimics the natural structure of the shoulder joint. The choice of implant will depend on local practice at recruiting sites but will include any anatomical shoulder implant from any manufacturer licensed for use in the UK. We will record and report the implants used. Due to its close relationship to normal shoulder anatomy, aTSR has the potential to provide patients with a return to a normal range of shoulder function; however, there is an associated risk of future revision due to failure of the rotator cuff.

##### Reverse total shoulder replacement

For the rTSR, the arrangement of the ball and socket component parts is reversed making use of the deltoid muscle for movement of the arm: it does not rely on an intact or functioning rotator cuff. The choice of implant will depend on local practice at recruiting sites but will include any reverse shoulder implant from any manufacturer licensed for use in the UK implanted using techniques consistent with manufacturer instructions. We will record and report the implants used.

### Post-operative care

Participants in both arms of the trial will be offered usual post-operative care including physiotherapy which can be delivered in person, remotely or using a hybrid model (as per usual care at participating sites). We will provide physiotherapists with the slides from a presentation given by a co-author at the 2022 British Elbow and Shoulder Society about the best current available evidence on rehabilitation after TSR. The timing and frequency of the physiotherapy will follow routine practice at participating sites. Where possible within the governance in place for each site, we will collect data including the content and number of physiotherapy sessions. We will also collect and summarise documents from participating sites that describe their typical post-operative rehabilitation protocols for participants having TSRs including exercise leaflets or templates.

### Outcomes

There is no consensus on the optimal outcome measures in shoulder arthroplasty, although Outcome Measures in Rheumatology Clinical Trials (OMERACT) has recommended four mandatory domains for trials of shoulder disorders: pain, function, global effect and adverse events.^13^ In choosing the primary outcome measure for this trial, we have followed the guidance of the Difference ELicitation in TriAls (DELTA) group and DELTA-2.^14^ The SPADI is very reliable, has low floor and ceiling effects, is valid for use in shoulder arthroplasty research and, as it is highly sensitive to change, it best enables us to answer the question of superiority of rTSR over aTSR in the two key domains identified by both the Patient and Public Involvement (PPI) group and OMERACT, namely pain and function.^15^ There is also a strong correlation between SPADI score and ROM.^16^ The Oxford Shoulder Score (OSS) is included as a secondary outcome to enable comparison of the trial population with the NJR dataset to explore external validity. The NJR has previously evidenced ceiling effects of the OSS in shoulder arthroplasty and lower responsiveness to change, making it unsuitable as the primary outcome instrument.^17^ NICE has recommended the primary outcome should be collected at 24 months post-surgery.^10^

#### Primary outcome

The primary outcome is the total pain and disability score measured using the SPADI at 24 months. The 13-item SPADI is a validated and sensitive instrument for use in TSR that assesses two domains: pain (five items) and functional activities (eight items) on numerical rating scales.^15 18^ The total score ranges from 0 to 100 where 0 indicates no shoulder pain or disability and 100 indicates worst possible shoulder pain or disability.

#### Secondary outcomes

- Combined pain and disability score: measured via the combined SPADI score at 3, 6, 12 and 18 months, and over 24 months.
- Individual pain and disability scores: measured via the subscale scores of the SPADI at 3, 6, 12, 18 and 24 months, and over 24 months.
- Pain and function: measured via the OSS, which is a 12-item patient-reported outcome measure of shoulder pain and function with five response categories and overall scale ranging from 0 (worst) to 48 (best).^19^ The OSS will be collected at 3, 6, 12 and 24 months.
- Global perceived effect: patient opinion about the change in their shoulder since the start of the trial will be assessed at 24 months via the question “Compared with just before the operation for your shoulder replacement at the start of the study, how would you say that your shoulder is now?”. Responses will be on a 5-point Likert scale with the following options: much improved, improved, same, worse and much worse.^20^
- Health-related quality of life: measured at 3, 6, 12 and 24 months via the EQ-5D-5L, a validated measure of health-related quality of life in terms of five dimensions (mobility, ability to self-care, ability to undertake usual activities, pain and discomfort, anxiety and depression) each with five levels of severity.^21^ The EQ-5D-5L will be used to calculate quality-adjusted life years (QALYs) according to NICE best practice guidance at the time of the analysis.
- ROM: The range of shoulder flexion, abduction, internal and external rotation will be assessed by a suitably trained blinded assessor at 24 months using a hand-held goniometer following trial specific instructions and recorded as continuous measurements except for internal rotation that will be assessed according to the position of the thumb to the spine.^22^
- Strength of shoulder: Shoulder strength will be measured at 24 months using a spring balance as described for the Constant Murley Score by a suitably trained blinded assessor.^22^ This will be done for both shoulders and repeated three times and will only be completed if the arm can be elevated to 90 degrees (abduction). Neither strength nor ROM will be collected during the interim follow-up timepoints given the need for in-person measurements by a trained team member. Both ROM and strength measurements will be collected only at baseline and at 24 months.
- Complications: Expected complications related to the affected shoulder will be recorded and will include (but are not limited to) deep and superficial wound infection (using Centers for Disease Control and Prevention definition),^23^ re-hospitalisation, and implant, nerve and skin problems. These complications will be recorded at 3, 6, 12 and 24 months. Complications specific to the arthroplasty implant (ie, glenoid loosening (keeled and pegged), scapula notching and lucency of the humeral stem)^24^,^26^ will be reviewed by the local surgeon using post-operative radiographs (typically anteroposterior and axial views) and 2 year radiographs (typically anteroposterior and axial views) or the most recent radiographs if not available at 2 years. This assessment will be performed using routinely taken radiographs and where feasible by a local surgeon who did not operate on the participant. Depending on the availability of funding, these radiographs will be pseudonymised of personal data and collected for central review independent of the surgeons at the local hospital.
- Re-operations: An operation to correct the complications of a previous operation due to, for example, an infection or dislocation. Re-operations will be recorded at 3, 6, 12 and 24 months.
- Revision and mortality: Rates of implant revision and patient mortality over the 24-month follow-up will be collected from hospital and the NJR records to identify patients in whom revision was undertaken elsewhere or death that is not recorded in the hospital records. The NJR definition of a revision will be used, which is any operation where one or more components are added to, removed or modified in a joint replacement or if a Debridement And Implant Retention with or without modular exchange is performed.^27^
- Resource use: Data on healthcare resource use will be collected to inform the economic evaluation (eg, length of hospital stay, re-hospitalisation, physiotherapy). Data will also be recorded about use of private care, days lost to work and normal activities. These data will be collected from participants and hospital records at 3, 6, 12 and 24 months.

##### Baseline data

The SPADI, OSS, EQ-5D-5L, ROM and shoulder strength will be collected at baseline by research teams at each site. Other validated measures at baseline will be a patient-reported 5-item frailty scale,^28^,^30^ a five-stage grading of muscular fatty degeneration,^31^ a co-morbidity index,^32^ an assessment of glenoid erosion via Walch’s classification^33^ and socio-demographic characteristics of participants. Ideally, shoulder ROM and strength will be collected before randomisation, along with other baseline data. However, in some cases, a blinded assessor may collect these data after randomisation (with the participant also blinded) as randomisation should occur ideally 2 weeks before surgery to allow for surgical planning and the patient may not be present at the time of randomisation.

### Participant timeline

**Figure 3:**
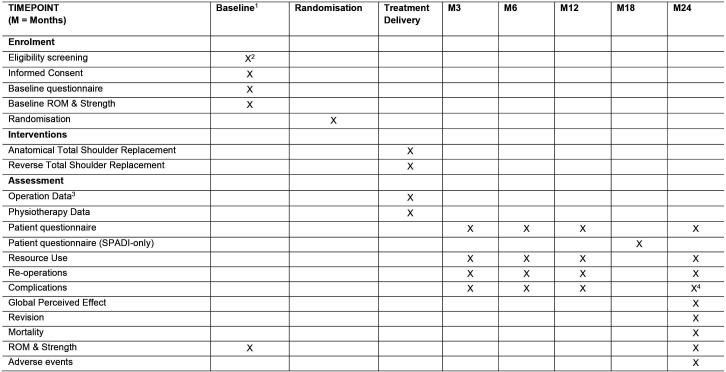
RAPSODI-UK trial assessment schedule. ROM, range of movement; SPADI, Shoulder Pain and Disability Index. ^1^Baseline assessments will be prior to randomisation except for baseline Range of Movement and Strength which may be collected, when necessary, after randomisation by an independent blinded assessor. ^2^This includes radiographs (typically anteroposterior and axial) to confirm osteoarthritis; CT or other imaging to assess glenoid erosion; and CT, MRI or US to assess the rotator cuff. ^3^Assessment of cuff integrity in theatre before operating and the possible use of fluoroscopy during surgery. ^4^This includes an assessment of implant problems using post-operative radiographs and radiographs at 24 months or earlier if not available. The radiographs will typically be anteroposterior and axial.

Figure 3 illustrates the overall schedule and flow of trial participants through the trial based on the recommended figure in the SPIRIT, from initial eligibility screening, consent, baseline data collection and randomisation, treatment delivery and follow-up data collection timepoints.

### Sample size

The primary outcome is the total SPADI score at 24 months. As our trial compares two active treatments, we expect a relatively small difference in SPADI score between groups at 24 months, so our target difference between the rTSR and aTSR groups is eight points (the smallest published, validated difference classed as clinically important).^34^ We also conservatively assumed a group SD of 25 points, based on three non-randomised studies.^35^,^37^ We plan to use a linear mixed-effects model in the primary outcome analysis, but due to a paucity of data in the literature to inform our between-measurement correlations, we instead calculate the sample size estimate based on an ANCOVA (‘baseline-as-a-covariate’) model.

Assuming a baseline/24-month SPADI score correlation of 0.35,^38^ 182 participants per group (364 total) are required to give 90% power to detect a difference of at least eight points (SD 25) between aTSR and rTSR, and a 5% significance level. Allowing for 15% loss to follow-up at 24 months,^39^ we aim to randomise a total of 430 participants.

The OSS is our key secondary outcome measure allowing direct comparison of the trial population to those in the NJR in order to explore external validity of the trial sample. Assuming a between-group difference of 2.7 points on the OSS, an expected SD of 8.80, and a baseline correlation of 0.35, the sample size of 430 would achieve 87.5% power using a similar ANCOVA analysis method and the same underlying assumptions.

### Data collection

Data will be collected from participants at baseline, 3-, 6-, 12-, 18- and 24-months post-randomisation. There will be a secondary outcome endpoint of 18-month follow-up to collect the SPADI only to try to help maintain trial engagement and reduce missing data of the primary outcome. Baseline data will be collected with participants at recruiting sites by a delegated member of staff, for example, research nurses. Follow-up data collection of the primary outcome and most secondary outcomes will be by postal questionnaire with supplemental telephone/video conference follow-up for non-responders or collected in clinic when attending at these timepoints as part of their routine care. Delegated research staff will also collect data from hospital records at 3, 6, 12 and 24 months. This includes a local assessment of routine post-operative radiographs and those routinely taken at 24 months (or earlier if not routinely available at this timepoint) to assess complications specific to the arthroplasty implant. Final follow-up data collection will include an outpatient clinic attendance at 24 months to assess shoulder ROM and strength by a blinded assessor. All reporting of data collection will be undertaken in line with the CONSORT statement.^12^

To minimise attrition, we will use multiple methods to maintain contact with participants, including full contact details (postal address, mobile phone number and email address if available). For postal data collections, two reminders will be sent to non-responding participants (at 2 and 4 weeks past the due date), with a final attempt to obtain data 6 weeks post-due date. Newsletters will be circulated to participants during the trial to keep them informed and engaged.^40^ Given that the 24-month follow-up clinic is not a routine appointment at all participating hospitals, all trial participants will receive a £20 gift voucher for attending the clinic and as a thank you for completing their follow-up questionnaires.

#### Data management

The participant questionnaires and hospital Case Report Forms (CRFs) will be designed using TeleForm software.^41^ The data collected by trial participants and sites using paper CRFs will be mailed (original paper CRFs) to York Trials Unit to be entered/scanned using TeleForm. When necessary, a site can securely return the CRF electronically. Participant questionnaires will be checked for missing data on receipt by York Trials Unit. In these instances, participants will be called to collect any missing primary outcome data and other missing data as feasible. Data collected via telephone or video call will be collected onto paper CRFs. As a duty of care, questionnaires will be checked immediately for anything that indicates that the participant could be at risk of harm. Where this occurs, the hospital team will be notified via email. To maximise data quality, key variables in the hospital CRFs will be reviewed by a York Trials Unit research data administrator for completion and accuracy, who will resolve any queries with staff at the relevant site. Following these initial checks, all CRFs will undergo a scanning process within TeleForm software, followed by second checking and validation against predetermined rules. A York Trials Unit data management system will be used to monitor CRF returns.

All data will be stored and transferred following York Trials Unit standard operating procedures. The staff involved in the trial (both at the sites and York Trials Unit) will receive training on data protection. The staff will be monitored to ensure compliance with privacy standards. Each site will hold data according to the General Data Protection Regulation as implemented in the Data Protection Act 2018.^42^ Essential trial documentation will be kept within the Trial Master File and Investigator Site Files. The Sponsor will ensure that this documentation is retained for a minimum of 5 years after the conclusion of the trial. All study-related information will be stored securely in the coordinating centre at the University of York. All electronic records will be stored on a password-protected server.

The Data Monitoring and Ethics Committee (DMEC) will be the only body to have access to the unblinded comparative data from the trial. The role of its members is to monitor these data and make any recommendations to the Trial Steering Committee (TSC) on whether there are any ethical or safety reasons why the trial should not continue. The TSC will provide overall supervision for the trial on behalf of the Sponsor and Funder.

#### Embedded Study Within A Trial (SWAT)

A SWAT will be conducted around participant retention with an embedded 1:1 RCT to investigate the impact of a newsletter sent 6 weeks prior to each of the 18- and 24-month follow-ups on completed SPADI follow-up rates at these timepoints (primary outcome 24-month SPADI completion), as a replication of registered SWAT 28.^40^

#### National joint registry linkage

Rates of implant revision and patient mortality at 24 months will be collected from hospitals and the NJR records to identify participants in whom revision was undertaken elsewhere (to the recruiting site) or death not recorded in the hospital records. An expression of interest has been registered with the NJR of England, Wales and Northern Ireland to embed the trial for collection of this data. Separate funding will be sought to investigate survival outcomes up to 5, 10 and 15 years in our trial participants.

In addition to the OSS, other characteristics of our trial population will be compared with the NJR cohort, such as age and gender. Participants will be asked if they agree to consent to complete questionnaires at future timepoints beyond the 24-month follow-up (subject to the progress of this trial and future funding) to further investigate long term outcomes.

### Statistical methods

Statistical analyses will be detailed in full in a Statistical Analysis Plan agreed by the independent DMEC prior to the end of data collection and are described in brief below.

Statistical analyses will be on an intention to treat (ITT) basis, with participants analysed in the groups to which they were randomised. Between-group treatment differences will be reported in the form of point estimates with 95% CIs and p values. Statistical significance will be declared at the 5% level, and analyses will be conducted in the latest available version of Stata or similar statistical software.

Baseline characteristics will be reported descriptively overall and by treatment group. Continuous data will be summarised as means, SD, medians and ranges as applicable, and categorical data as frequencies and percentages. All trial outcomes will be reported descriptively by group at all time points at which they were collected.

#### Primary outcome

The primary comparison of interest is the between-groups difference in SPADI score at 24 months. The estimate for this difference will be derived from a constrained longitudinal data analysis model.^43^ This will be a linear mixed-effects model, featuring SPADI score at baseline, 3-, 6-, 12-, 18- and 24-months post-randomisation as the outcome, intervention group, timepoint, age and gender as fixed effects, and participant identifier and trial site as random effects. A series of group-by-time point interaction effects will be included as fixed effects, thereby making no assumptions about the shape of the SPADI score trajectory over time. The model will be constrained so that the baseline SPADI scores are equal between groups.^43^ The model will use maximum likelihood estimation, with an unstructured covariance matrix. The between-groups difference in SPADI score at 24 months will be extracted from this model as the primary outcome and reported with a 95% CI and p value.

#### Secondary outcomes

The between-group differences for total SPADI score at 3-, 6-, 12- and 18-months, and over 24 months will also be extracted from the primary analysis model as secondary outcomes. The other secondary continuous outcomes (eg, SPADI pain and function subscale scores, OSS) will be analysed using an identical mixed-effects model with the same covariates and covariance structure. While also a continuous secondary outcome, the EQ-5D-5L will exclusively be used in the health economic evaluation.

ROM and strength are collected at two timepoints (baseline and 24 months). The 24-month outcomes for these measures will be compared via a mixed-effects linear regression model with intervention group as the predictor variable, as well as age, gender and baseline value of the outcome, and site as a random effect.

The ordinal outcome of the global perceived effect outcome at 24 months will be analysed using mixed-effects ordinal logistic regression, adjusting for age, gender and baseline combined SPADI score, with site as a random effect. Time-to-event outcomes (time to revision/re-operation/death) will be explored by comparing restricted mean survival times between intervention groups. Safety outcomes (adverse event rate, complication rate and types of complication) will be summarised across all timepoints at which they are collected.

#### Subgroup analyses

Age is considered a key moderating factor on the effects of recovery from TSR^44^ and it informs surgeons’ clinical decisions about whether to perform aTSR or rTSR. We therefore will directly compare aTSR and rTSR in two specific subgroups, for differential treatment effects: age 60–69 years (where the two surgical approaches are carried out in approximately even numbers in routine practice), and 70+years (where clinically, rTSR is generally given more commonly than aTSR). We shall include an interaction term between age and treatment group in the primary analysis model and shall also model the primary outcome in each of the two subgroups separately. We anticipate that rTSR will have increased effectiveness over aTSR in participants aged 70+years than in younger patients.

#### Additional analyses

There is the potential for participants in this trial not to receive their allocated intervention (eg, surgery not performed, or they cross over from one treatment arm to the other). A complier average causal effect (CACE) analysis will be carried out at the 24-month time-point to account for this. Compliers are defined as participants who received their allocated TSR. The CACE analysis will be implemented using a two-stage least squares instrumental variable approach with randomised treatment assignment as the instrument variable and the received allocated TSR as the exogenous variable.^45^ The linear regression model will control for age, gender and baseline total SPADI score.

#### Missing data

The primary analysis model assumes missing outcome data are missing at random (MAR) and uses full information maximum likelihood to implicitly handle missing outcome data. It is expected that other covariates included in the model, for example, gender, are unlikely to be missing. Therefore, as it is unlikely to have a large proportion of missingness in the analysis, multiple imputation will not be performed as part of the primary analysis. However, it is possible that participants who failed to complete their follow-ups will differ from those who did (for example, had worse pain/disability following surgery and therefore would have scored higher on the SPADI if they had completed the follow-up). This would suggest the data were missing not at random and represent a departure from the MAR assumption. Additional sensitivity analyses will be conducted using a pattern-mixture model to examine how sensitive the primary treatment effect estimate is to an assumption that (a) all of these missing observations (if present) are poor outcomes in the SPADI, and conversely that (b) all of these missing observations are beneficial outcomes in the SPADI, and thereby modelling ‘best case’ and ‘worst case’ scenarios for the primary outcome analysis.

While the results of this sensitivity analysis will not be directly comparable to the primary analysis model, it will be able to give an indication of how sensitive the estimate of the treatment effect is to departures from the MAR assumption in the primary outcome data.

### Cost-effectiveness analysis

A comprehensive economic evaluation will be conducted to assess the cost-effectiveness of rTSR compared with aTSR. First, a within-trial analysis over the trial’s 24-month time horizon will evaluate the short-term cost-effectiveness of the two surgical approaches. Second, a decision-analytic model is proposed to extrapolate results beyond the trial period and evaluate the longer-term cost-effectiveness, provided sufficient data inputs are available to support robust modelling.

The cost-effectiveness analysis will follow NICE Health Technology Evaluations: The Manual^46^ and Decision Modelling for Health Economic Evaluation.^47^ The primary analysis will be conducted from an NHS and personal social services perspective, with a 24-month time horizon. Healthcare resource use, including that associated with the original shoulder surgery, adverse events, revisions and post-operative rehabilitation, will be used alongside published national unit costs and other sources to estimate the costs associated with both surgical approaches.^48^ The EQ-5D-5L will be used with an area under the curve approach to estimate QALYs accrued during the trial.^21 49 50^

Regression analysis, adjusted for key covariates, will be used to estimate net costs and QALYs by TSR allocation on an ITT basis. These will be combined to calculate the incremental cost-effectiveness ratio (ICER), expressed as the cost per QALY gained. A non-parametric bootstrapping resampling technique will be used to explore uncertainty and estimate the probability that rTSR is cost-effective compared with aTSR at different willingness-to-pay thresholds.^51 52^

To account for missing data, Rubin’s multiple imputation techniques will be used to impute missing observations, and the imputed dataset will form the basis of the primary analysis.^53^ Specifically, multiple imputation by chained equations will be used to impute missing outcomes with 25 imputations, under the assumption that the missing outcome data are missing at random.^54^ As part of the sensitivity analysis, we will also conduct a complete-case analysis, including only participants who have complete cost and QALY data. In addition, a secondary analysis from a wider societal perspective will be conducted to account for indirect costs associated with participants’ shoulder condition and the trial interventions, beyond the healthcare costs captured under the NHS and personal social services perspective in the primary analysis. This secondary analysis will include productivity losses arising from missed days of paid employment and time unable to perform usual unpaid activities, as well as any private healthcare costs incurred.

A decision-analytic model is planned to explore the long-term cost-effectiveness of the two surgical procedures beyond the trial period. This model will incorporate trial-collected data and information from relevant literature, including revision rates derived from the NJR. The feasibility of the model will depend on the availability of sufficient data inputs for a robust and reliable analysis.

### Nested qualitative study of patient experience

Semi-structured qualitative interviews will be conducted with a subsample of trial participants at approximately 2 and 12 months after surgery. These time points have been selected as they are important in the recovery process: at 2 months, patients are anticipated to begin regaining use of their arm and at 12 months functional recovery is starting to plateau for most individuals.^55 56^

We aim to recruit approximately 20 individuals (approximately 10 from each of the two surgical approaches). This will provide approximately 40 interviews (two per person). A high level of detail and depth is expected within the data set. We anticipate that this sample size will be sufficiently large to yield a range of perspectives, while also being small enough to be effectively managed and analysed, producing a rich understanding of individuals’ experiences. Purposive sampling will be conducted to ensure approximately equal numbers from each of the two surgical approaches, and to ensure variation in age (individuals aged 60–69 and 70+) and geographical location (a minimum of four trial sites). We will also aim to include variation in gender and ethnicity within the sample. We will sample participants to interview from those who have variation in pain and function scores, where possible. If the initial sample lacks such variation, the qualitative team may recruit additional participants at 12 months, using SPADI data to inform sampling.

Interviews will be conducted by telephone or video call, and topics will include patients’ priorities and expectations, experiences of recovery (including pain and functioning) and patients’ acceptability of their TSR (see RAPSODI-UK Qualitative interview topic guide for full interview schedule). The interview schedule was informed by the PPI group, the research literature and the Theoretical Framework of Acceptability.^57^

An inductive, data-driven, thematic analysis will be conducted using the Framework approach to identify and understand patterns in the data.^57 58^ Framework provides a transparent and systematic approach to structuring a thematic analysis. Matrices are used to summarise data. Analysts use these matrices to make sense of and interrogate the data, examining issues both within and across participants. The matrices can be organised to enable examination of data by groupings—such as by timepoint of interview (2 months or 12 months), or by participants’ surgical intervention (aTSR or rTSR). In an iterative process, preliminary findings will be shared with PPI contributors and clinical research team members to gain and incorporate their insights into the analysis. Possible similarities and differences in experience by allocated surgical approach will be explored to help explain the main trial findings.

### RAPSODI-Australia trial collaboration

The RAPSODI-UK trial group are collaborating with a team leading a parallel trial in Australia (RAPSODI-Australia), funded by the National Health and Medical Research Council . Australia’s national growth in shoulder replacement surgery over the last 12 years is among the highest globally with a 338% increase since 2008.^59^ A similar number of replacements is undertaken annually compared with the UK, despite the smaller Australian population. RAPSODI-Australia is currently underway, collecting the same dataset. This will enable future data pooling, secondary analyses and moderator analyses. We also plan to share the pseudonymised qualitative data collected from the two countries to further explore similarities and differences in patient experience.

### Monitoring

Wrightington, Wigan and Leigh Teaching Hospitals NHS Foundation Trust is the Sponsor for this trial and takes overall responsibility for the quality of trial conduct. This trial will be fully compliant with the Research Governance Framework and Medical Research Council GCP Guidance. The coordination of the RAPSODI-UK trial will be managed by York Trials Unit in collaboration with the Sponsor and joint chief investigators (IT and JD). The Trial Management Group (TMG) will monitor the day-to-day management of the trial. The TSC will monitor the progress of the trial, provide independent advice and the independent chair will make recommendations to the funder. The independent DMEC will monitor the data arising from the trial and make recommendations to the TSC about trial continuation based on ethical and safety considerations. The trial will also be monitored by the Sponsor and a representative will be invited to attend the TMG, TSC and DMEC.

York Trials Unit is experienced in working with local investigators at recruitment sites to ensure ethical and efficient delivery of trials in compliance with the trial protocol. This will include undertaking remote monitoring of participating hospitals to ensure integrity of the trial. In addition to regular TMGs, the trial team will keep in regular contact with sites, newsletters and other forms of communication to monitor progress, support low-recruiting sites and to share good practice across all sites.

### Protocol modifications

Any substantial amendments that affect the scientific value or conduct of the trial will be submitted to Research Ethics Committee (REC) for approval, having been discussed with the TMG and agreed with the Funding Body, Sponsor, TSC and DMEC. Any minor modifications to the protocol will be agreed with the Sponsor before submission for approval to REC. All amendments, whether substantial or not, will be listed in the published Final Report to the Funding Body.

### Adverse event reporting and harms

Adverse events (AEs) will be defined as any untoward medical occurrence in a trial participant to whom a treatment has been administered, and which does not necessarily have a causal relationship with the treatment. Only medical occurrences related to treatment for the shoulder condition that are ‘unexpected’ and up until the 24-month follow-up will be classified as events when non-serious. This is because ‘expected’ events are well known complications for the two routine treatment options which the specialist clinical care teams will be experienced in managing.

Serious adverse events (SAEs) will be defined as any untoward medical occurrence that:

- Results in death.
- Is life-threatening (that is, it places the participant, in the view of the Investigator, at immediate risk of death).
- Requires hospitalisation or prolongation of existing inpatients’ hospitalisation (unplanned refers to emergency hospitalisations resulting in an inpatient stay; prolonged hospitalisation is deemed to be where a patient’s stay is longer than expected).
- Results in persistent or significant disability or incapacity.
- Any other important medical condition which, although not included in the above, may require medical or surgical intervention to prevent one of the outcomes listed.

SAEs that may be expected as part of the surgical interventions and that do not need to be reported to the main REC include: complications of anaesthesia or surgery (eg, wound complications, infection (superficial and deep), damage to a nerve or blood vessel and thromboembolic events), skin problems, implant problems (eg, fracture, rotator cuff tear and instability) and secondary operations for or to manage instability, infection, fracture, non-union or for symptoms related to the prosthesis.

Medical occurrences that are serious and about treatment for the shoulder condition and up until the 24-month follow-up will all be reported as SAEs (including deaths for any reason) whether expected or not. Ongoing review of AEs will take place during regular TMG meetings, discussed with the TSC and DMEC and reported to the Sponsor and research ethics committee in line with their guidelines. Causality and expectedness of SAEs will be confirmed by either of the joint CIs, and any SAEs that are deemed to be unexpected and related to the trial will be notified to REC and the Sponsor. Follow-up reports a month later of all AEs and SAEs will be reviewed by either of the joint CIs to ensure that adequate action has been taken and progress made.

### Auditing

A statement of permission to access source data by study staff and for regulatory and audit purposes will be included within the participant consent form with explicit explanation as part of the consent process and participant information sheet. York Trials Unit will permit authorised representatives of the Sponsor and applicable regulatory agencies direct access to source data to conduct trial-related monitoring, audits and regulatory inspection. Trial participants are informed of this during the informed consent discussion. Participants will consent to provide access to their medical notes.

On the analysis and publication in scientific journals, the anonymised trial data will be available for other researchers on reasonable request to the Chief Investigators (Ian.Trail@wwl.nhs.uk and jd96@leicester.ac.uk). Requests will be considered on a case-by-case basis by the TMG and managed according to York Trials Unit, University of York processes and procedures.

### Patient and public involvement

RAPSODI-UK has been developed with patient advisors who have had TSRs, as well as with members of the public as part of the PPI group. The PPI group will meet regularly during the set-up phase of the trial and will continue to be involved during its conduct to support the development of patient-facing documents, advise on trial processes and suggest how best to report trial findings to the public and patients. We aim to gain valuable feedback about patient-facing materials during these PPI meetings, and as a result, patient newsletters and posters will be amended accordingly and continually improved. Newsletters designed specifically for the PPI group will be circulated to keep the members informed of study progress. To ensure ongoing oversight, two independent lay members who have received TSRs will attend the approximately biannual TSC meetings. Both individuals, as well as the PPI group based at the Sponsor, will play an important role in developing easily understandable key messages about trial findings for our dissemination strategy.

## Ethics and dissemination

### Research ethics approval

This trial protocol has been reviewed and approved by the London - Queen Square Research Ethics Committee, Rec Reference 22/LO/0617 on 4 October 2022 and has been registered at ISRCTN12216466. The current protocol version is 4.0 (16 October 2024).

### Dissemination policy

The research team will produce lay summaries targeted at specific stakeholders, presentations at relevant professional conferences and press releases through the collaborating NHS organisations and universities. A plain language summary will be disseminated to those trial participants who expressed an interest in learning about the trial findings.

Dissemination will focus on supporting the widespread implementation of the superior treatment in NHS sites (if superiority is statistically confirmed). Articles for publication in peer-reviewed journals will be produced, irrespective of the trial outcome. Data will be made available to allow for inclusion in future meta-analyses with studies of the same treatments from other trials and will also be used for the merging of data between the two trials being undertaken in the UK and Australia.

## Supplementary material

10.1136/bmjopen-2025-106740 online supplemental file 1

10.1136/bmjopen-2025-106740 online supplemental file 2
