# Supplementary material for: Anatomic versus reverse total shoulder replacement for patients with osteoarthritis and intact rotator cuff: the RAPSODI-UK randomised controlled trial protocol
Source: BMJ Open. 2025 Dec 12;15(12):e106740. doi: 10.1136/bmjopen-2025-106740 (PMC12706210; doi:10.1136/bmjopen-2025-106740)
Supplement: online supplemental file 1 [file bmjopen-15-12-s001.docx]

### Supplementary File

### Internal pilot

We will select a minimum of 28 high and medium volume hospital sites and prioritize sites that have performed at least 130 shoulder arthroplasties in the three years to 2019. Based on NJR data for 2019, the 28 sites perform approximately 1,054 TSRs per year for shoulder OA. Assuming 60% of patients’ rotator cuffs are intact, a conservative estimate based on the proportion of patients from the NJR with a diagnosis of OA having aTSR or hemiarthroplasty, and a predicted minimum 55% consent rate, we aim to recruit 347 participants per year (an average 1.03 per site per month) once all sites are open to recruitment. We will set up sites in a staggered formation, prioritising the high-volume sites, resulting in an expected recruitment rate of 1.06 per site per month during the internal pilot phase. We aim to reach the target sample size over a 24-month recruitment period. The Trial Management Group (TMG) will regularly monitor the number screened, eligible, approached and randomised and take appropriate mitigating action, including recruiting additional sites (up to a maximum of 35), as necessary.

Recruitment and monitoring data will be discussed with the Independent Data Monitoring Committee (IDMC) and the Trial Steering Committee (TSC) who will recommend to the funder about proceeding to the main trial. The pilot will last 1/3 of the recruitment period (8/24 months) and aim to recruit 1/5 (20%) of the total target (n=86/430), due to the large number of sites and staggered set-up, and a target to set up 16 of the overall target of 28 sites. A proportion of recruits (we estimate up to 5%) may not have an intact rotator cuff when visually inspected at the time of surgery, despite appearing intact on pre-operative imaging. When this occurs patients randomised before the planned operation to aTSR would cross-over to rTSR, whereas those randomised to rTSR would proceed as allocated. This could lead to unbalanced cross-over. This risk could be mitigated by randomising at the time of surgery but this would be more challenging for the surgical team to be prepared to undertake either intervention and have both prosthesis kits available for use and may increase theatre time. We therefore plan to randomise before surgery. The number and proportion of participants, in both groups, seen not to have an intact rotator cuff during surgery, and the resulting number of cross-overs in the aTSR group, will be assessed during the pilot phase to inform whether to continue or not with randomisation before the planned surgery. We may need to continue to monitor this beyond the pilot depending on whether there are sufficient patients to make a decision, also as new sites are enrolled it will be important to monitor that this does not change. Any patients who are identified as having non-intact rotator cuff during surgery will remain in the trial and be followed-up and analysed following the principles of intention-to-treat. We will also monitor, in both groups, whether there are cancellations or postponements in surgery post-randomisation, and the time between randomisation and surgery. Routine imaging done within six months before surgery will be used, or earlier imaging if not routinely available, to assess the integrity of the cuff to reduce the risk of time dependent changes in cuff status.

Progression criteria for recruitment will be: Green (recruit ≥100% of target [n≥86]); Amber (60%-99% [n=52-85]), review and implement methods to increase recruitment, if feasible, otherwise stop trial); Red (<60% of target [n<52]) stop unless mitigating circumstances. This and other progression criteria are detailed in Table 1.

Table 1: Progression criteria for the internal pilot

|  | ***Red*** | ***Amber*** | ***Green*** |
| --- | --- | --- | --- |
| *Total number of participants recruited* | *<52* | *52-85* | *≥86* |
| *Recruitment rate/site/month* | *<0.64* | *0.64-<1.06* | *≥1.06* |
| *Number of sites opened* | *<10* | *10-15* | *≥16* |
| *Percentage randomised to aTSR that receive it* | *<70%* | *70-<95%* | *≥95%* |

Screening logs will be used to monitor the number screened, eligible, approached and randomised. The extent to which eligible patients are not given the opportunity to participate in the trial and whether there are any trends in the characteristics of patients not approached will be monitored. We will also monitor the reasons why patients decline participation in the trial (if they agree to provide this information). We will explore whether any factors under our control can be addressed during the pilot.

The age cut-off of 60 years in our patient population is consistent with the threshold for equipoise for consideration of rTSR amongst clinicians which was informed from a survey of surgeons when designing the study. Below this age there are concerns that the patient will require revision of the implant during their lifetime and that this may be more complicated after rTSR than aTSR. We will monitor the proportion of the sample that are in the 60-69 age group and above using the screening logs. We will closely monitor if the recruited patient group is similar in characteristics to the population included in the NJR from data published in their annual report. We will use this information to inform the ongoing support and training provided by York Trials Unit (YTU) to recruitment staff at individual sites and at cross site meetings to share good recruitment practice from other orthopaedic surgical trials.

At the end of the pilot phase, data required to assess the trial against the pre-specified progression criteria will be summarised descriptively. No formal hypothesis testing will be undertaken, nor will this involve looking at any primary or secondary outcome data. The IDMC and TSC will review progress and recommend that the trial continue without amendments, continue with major/minor amendments, or discontinue.
