# Supplementary material for: Anatomic versus reverse total shoulder replacement for patients with osteoarthritis and intact rotator cuff: the RAPSODI-UK randomised controlled trial protocol
Source: BMJ Open. 2025 Dec 12;15(12):e106740. doi: 10.1136/bmjopen-2025-106740 (PMC12706210; doi:10.1136/bmjopen-2025-106740)
Supplement: online supplemental file 2 [file bmjopen-15-12-s002.docx]

**RAPSODI-UK Interview Topic Guide**

*This topic guide summarises the main areas to be explored for each interview. As with any qualitative interview, these headings are intended as a starting point to ensure the primary issues are covered, whilst allowing flexibility for new issues to be explored. This topic guide may be amended as interviews progress e.g. should topics be raised by participants which researchers had not anticipated, those topics could be added to the topic guide so that later participants are asked about those issues.*

***2 MONTH POST SURGERY INTERVIEW***

**Main interview**

*Before surgery:*

- Please can you tell me a bit about how your shoulder affected you before surgery?
- How, if at all, did your shoulder affect your sense of self?
- *Areas to explore within this:*
  - Pain
    - Pain (in general life, when pushing, when reaching for something, when lying on it)
    - Being able to move your arm or your shoulder
  - Daily living
    - How, if at all, did your shoulder affect your relationships with others? E.g., interactions with family/friends, colleagues, others?
    - How, if at all, did your shoulder affect your roles in life? E.g. social/community/work roles and responsibilities? Caring roles?
    - Home activities (being able to cook and clean, eating, doing the shopping, carrying food)
    - Getting washed and dressed (activity limitations in terms of washing hair, back, washing self, getting dressed - doing up buttons, pulling on trousers, brushing hair etc)
    - Hobbies
    - Using transport or getting in or out of a car
    - Sleep (difficulty getting comfortable, pain in bed)
    - Intimacy
  - Work and financially
    - At work (paid or volunteer) - (general movement, carrying heavy objects)
    - Financial impact
  - Emotionally – emotional well-being

*Note to interviewer: check that participants have had the opportunity to consider the following areas: Impairment (e.g. pain, ability to move arm/shoulder), Activity limitations (e.g. unable to get dressed) and Participation restrictions (e.g. unable to work, unable to socialise with friends).*

- What made you decide to have surgery?
- What did you hope to gain by having surgery?

*Since surgery:*

- Please can you tell me a bit about how you have been getting on since your shoulder replacement? *Alternative phrasing:* How are you finding the recovery process?
- How, if at all, has your shoulder been affecting your sense of self?
- *Areas to explore within this:*
  - Pain
    - Pain (in general life, when pushing, when reaching for something, when lying on it)
    - Being able to move your arm or your shoulder
  - Daily living
    - How, if at all, has your shoulder been affecting your relationships with others? E.g. interactions with family / friends, colleagues, others?
    - How, if at all, has your shoulder been affecting your roles in life? E.g. social/community/work roles and responsibilities? Caring roles?
    - Home activities (being able to cook and clean, eating, doing the shopping, carrying food)
    - Getting washed and dressed (activity limitations in terms of washing hair, back, washing self, getting dressed - doing up buttons, pulling on trousers, brushing hair etc)
    - Hobbies
    - Using transport or getting in or out of a car
    - Sleep (difficulty getting comfortable, pain in bed)
    - Intimacy
  - Work and financially
    - At work (paid or volunteer)- (general movement, carrying heavy objects)
    - Financial impact
  - Emotionally – emotional well-being

*Note to interviewer: check that participants have had the opportunity to consider the following areas: Impairment (e.g. pain, ability to move arm/shoulder), Activity limitations (e.g. unable to get dressed) and Participation restrictions (e.g. unable to work, unable to socialise with friends).*

- Are there activities that you’re not able to do at the moment that you think you will be able to do when you’re fully recovered?
  - *Follow-up: ask whether this was something they used to do – e.g. how long ago?*
- What activities would you *like* to be able to get back to doing that you’re not able to do at the moment? *Additional phrasing: Is there anything that you used to do before your shoulder problem that you would like to be able to get back to doing?*
  - Is this something you think you will be able to manage in the future?
  - How long do you think it might take to be able to …?
- Do you have any strategies in place to help you get back to this/these activities?
- Are you concerned about being able to do anything in the future?
- Is there anything that you think is making your recovery more difficult?
  - *Prompt*: is there anything you think could help your recovery?

*Acceptability of anatomic shoulder replacement and reverse shoulder replacement and treatment received:*

- How do you think a shoulder replacement works? How do you think it helps people like you with shoulder problems?
- Can you tell me what you remember being told about the two different surgeries - total (anatomic) shoulder replacement and reverse shoulder replacement? *How were you told about this? Did you feel well informed?*
- Thinking about the two different types, how do you think each one works? (*Prompt*: How do you think the anatomic shoulder replacement surgery works? How do you think reverse shoulder replacement surgery works?)
- Thinking back to before your surgery, how did you feel about the idea of having a shoulder replacement?

*Prompts*: How comfortable did you feel about the idea of receiving a total shoulder (anatomic) replacement? What about the reverse shoulder replacement?)

- How do you feel about your shoulder replacement now?
- If we went back in time to before your operation, would you be happy to have the same surgery again? Why? /why not? Prompt: Would you recommend the surgery you had to others?
- How effective do you think your shoulder replacement has been so far for dealing with your shoulder difficulties?
- Thinking back to before your operation, when discussing the two types, how effective did you think each type would be?
- How much of a burden has having surgery been for you? (*prompt*: how much effort has it taken?)
- Thinking about the two types of surgery, do you think there would be any difference in burden between these? Why/why not?
- How much do you feel having surgery has interfered with your other priorities?
- Thinking about the two types of surgery, do you think there would be any difference between them in how they might interfere with your priorities?
- Thinking back to before your operation, how confident did you feel about managing your shoulder replacement surgery?
- Thinking about the two types of surgery, was there any difference in how confident you felt about managing surgery?

*About the RAPSODI study: Acceptability and understanding of study and recruitment*

- How did you find the process of being asked to take part in the RAPSODI trial?
  - Did you receive a patient information leaflet? Was this easy to understand? Could anything be improved to make it clearer? How did you find the amount of information you received?
  - Who approached you to take part in the study? How did they explain this to you?
  - When were you asked to take part? Was this a good time?
  - Was anything not explained to you very well? Could anything have been better or clearer? Was there anything we did not tell you that you think we should tell patients in the future?
- Is there anything about taking part in the study that you have found difficult?
- Why did you decide to take part? Has this been worthwhile?
- Can you tell me what you understand when I say that clinical trials often involve a process of participant randomisation? Have you heard about randomisation before?
  - Do you understand why you have not been told what surgery you had? (remind patient that researcher doesn’t know either).
  - How did you feel about being allocated to a type of surgery through randomisation?
- How have you been finding completing the study questionnaires?
  - Was there anything you have found difficult with them?
  - Needed support with?

*Before we finish…*

- Are there any other issues or questions you would like to discuss?
- We would like to ask you some general questions about yourself before we finish. Would that be ok?
  - What is your age?
  - What is your gender?
  - What is your ethnicity?
  - What is your postcode?

**End of interview**

- Thank participant and ask if they have any comments.
- Explain again about how data will be used and reiterate about anonymity and confidentiality.
- Provide opportunity for questions and state that the lead researcher is contactable after the interview, should questions arise.

***12 MONTHS POST SURGERY INTERVIEW***

**Main interview**

*About you:*

- Please can you tell me a bit about how you have been getting on since our last interview. *Prompt*: How have you been finding the recovery process since our last interview? Have you experienced any particular difficulties?
- How, if at all, has your shoulder been affecting your sense of self?
- Areas to explore within this:
  - Pain
    - Pain (in general life, when pushing, when reaching for something, when lying on it)
    - Being able to move your arm or your shoulder
  - Daily living
    - How, if at all, has your shoulder been affecting your relationships with others? E.g. interactions with family / friends, colleagues, others?
    - How, if at all, has your shoulder been affecting your roles in life? E.g. social/community/work roles and responsibilities? Caring roles?
    - Home activities (being able to cook and clean, eating, doing the shopping, carrying food)
    - Getting washed and dressed (activity limitations in terms of washing hair, back, washing self, getting dressed - doing up buttons, pulling on trousers, brushing hair etc)
    - Hobbies
    - Using transport or getting in or out of a car
    - Sleep (difficulty getting comfortable, pain in bed)
    - Intimacy
  - Work and financially
    - At work (paid or volunteer) - (general movement, carrying heavy objects)
    - Financial impact
  - Emotionally – emotional well-being

*Note to interviewer: check that participants have had the opportunity to consider the following areas: Impairment (e.g. pain, ability to move arm/shoulder), Activity limitations (e.g. unable to get dressed) and Participation restrictions (e.g. unable to work, unable to socialise with friends).*

- Do you feel as though you are fully recovered now?
- Are there activities that you’re not able to do at the moment that you think you will be able to do when you’re fully recovered?
  - *Follow-up: ask whether this is something they used to do – e.g. how long ago?*
- What activities would you *like* to be able to get back to doing that you’re not able to do at the moment? *Additional phrasing: Is there anything that you used to do before your shoulder problem that you would like to be able to get back to doing?*
  - Is this something you think you will be able to manage in the future?
  - How long do you think it might take to be able to …?
- Do you have any strategies in place to help you get back to this/these activities?
- Are you concerned about being able to do anything in the future?
- Is there anything that you think is making your recovery more difficult?
  - *Prompt*: is there anything you think could help your recovery?

*Acceptability of anatomic shoulder replacement and reverse shoulder replacement and treatment received:*

- How do you feel about your shoulder replacement now?
- If we went back in time to before your operation, would you be happy to have the same surgery again? Why? /why not? Prompt: Would you recommend the surgery you had to others?
- How effective do you think your shoulder replacement has been so far for dealing with your shoulder difficulties?
- How much of a burden has having surgery been for you? (*prompt*: how much effort has it taken?)
- How much do you feel having surgery has interfered with your other priorities?

*About the RAPSODI study: Acceptability and understanding of study and recruitment*

- Since we last spoke, is there anything about taking part in the study that you have found difficult?
- How have you been finding completing the study questionnaires?
  - Was there anything you found difficult with them?
  - Needed support with?
- Has taking part been worthwhile for you?

*Before we finish…*

- Are there any other issues or questions you would like to discuss?

**End of interview**

- Thank participant and ask if they have any comments.
- Explain again about how data will be used and reiterate about anonymity and confidentiality.
- Provide opportunity for questions and state that the lead researcher is contactable after the interview, should questions arise.
