# Supplementary material for: Anatomic versus reverse total shoulder replacement for patients with osteoarthritis and intact rotator cuff: the RAPSODI-UK randomised controlled trial protocol
Source: BMJ Open. 2025 Dec 12;15(12):e106740. doi: 10.1136/bmjopen-2025-106740 (PMC12706210; doi:10.1136/bmjopen-2025-106740)
Supplement: Supplementary file 3 [file bmjopen-2025-106740.reviewer_comments.pdf]

PEER REVIEW HISTORY

BMJ Open publishes all reviews undertaken for accepted manuscripts. Reviewers are asked to complete a checklist review form and are provided with free text boxes to elaborate on their assessment. These free text comments are reproduced below.

ARTICLE DETAILS

Title (Provisional)

Anatomic versus reverse total shoulder replacement for patients with osteoarthritis and intact rotator cuff: the RAPSODI-UK randomised controlled trial protocol

Authors

Rodrick, Hannah L; Dias, Joseph; Watts, Adam C; Walton, Michael J.; Brealey, Stephen; Page, Richard; Foster, Nadine E.; Boland, Katy; Cunningham, Lindsay J.; Fairhurst, Caroline; Geoghegan, John; Greenwood, William; Hewitt, Catherine; Kirwan, Cliona; Leggett, Heather; McDaid, Catriona; Parkes, Matthew; Parrott, Steve; Powell, Rachael; Quicke, Jonathan G.; Roberts, Gareth; Rose, Fiona; Singh, Harvinder Pal; Spickett, Helen; Sutton, Chris J.; Wiggins, Fraser; Wu, Qi; Trail, Ian

---

VERSION 1 - REVIEW

**Reviewer**	**1**
**Name**	**Launonen, Antti**
**Affiliation**	**Tampere University Hospital**
**Date**	**06-Aug-2025**
**COI**	**None**

Anatomic versus reverse total shoulder replacement for patients with osteoarthritis and intact rotator cuff: the RAPSODI-UK randomised controlled trial protocol

Thank you for the opportunity to review this necessary RCT. I think we need to investigate the limits to fill our knowledge gaps and push the boundaries. This trial is one of those important ones that help us choose the best treatment methods in the future.

Even meticulously written, I would like to give some perspective and suggestions to consider in (hopefully) improving the trial. I acknowledge the sister trial in Australia has already started, and this one should be similar in main points.

The foremost concern I think should be addressed, either by discussing or creating an analysis around it, is the rTSA patients who are in high demand. E.g. construction workers or very fit patients going to the gym (lifting 100+kg), near 60 years old. At least in the North, we give rTSA patients an absolute lifting limit of max 10kg after the surgery. Naturally, that is something out of the blue – not investigated in any way, just as a mechanical consideration.

However, that should be addressed in some way within the protocol, especially when the patients are also blinded to the allocation. For me, the optimal solution would be to analyse the result, if occupation, patient fitness or hobbies have affected loosening or revision rate. Or then just have a cutoff and exclude such patients.

The introduction is a bit lengthy. I think PPI could be placed in another part of the manuscript, and some elements of the introduction could be used in a discussion, which is now lacking. In the discussion, there could be more considerations of different patient populations and safety.

In cost-effectiveness analysis, indirect costs could be included, if possible. As mentioned earlier, the patients at work may be most affected by different types of prostheses or may not be able to continue in their work. This would be an essential aspect as well.

There are some points in the inclusion criteria;

As there are no formal indications for the OA surgery in the shoulder, I think the degree of OA should be recorded and analysed between the two groups to maintain similar conditions.

US is very low in specificity to detect the cuff rupture, especially in patients with advanced OA. I am not sure if it's an eligible way to inspect the cuff integrity within the trial.

"Minimal glenoid erosion" is very shady and may be different from clinician to clinician even in a pragmatic setting. I would also add an analysis of the degree, with e.g., Walch classification.

P10, l 20 Not absolutely sure, but according to GCP, a signed consent should be sought in all times? Patients should be present in the treatment negotiation, so it should be possible?

What comes to outcomes, did you consider WOOS? There are some clinical registry data available around WOOS in the NARA registry, so the trial outcomes could be mirrored with registry data.

The primary outcome time is from randomization. Why not from the treatment? I think the waiting list from randomization to surgery may be lengthy in some instances, so the actual outcome time is less than two years.

I am not familiar with Teleform software, but from the protocol, I understand you are managing papers. Why not use something like Redcap or a similar tool? I have had a hard time collecting all the paperwork of 250 patients, and it is very time-consuming, and human error possibilities are very high, resulting in the loss of some patient data. After going all electronic, the data is secured once and for all. No missing data, and you can see the progression of the trial by checking the main view from the program desktop.

The statistical methods section is quite brief. Will you create a statistical analysis plan? Please mention it if that will be done.

**Reviewer**	**2**
**Name**	**Ramadanov, Nikolai**
**Affiliation**	**University Hospital Brandenburg/Havel Brandenburg Medical School**
**Date**	**01-Sep-2025**
**COI**	**None**

1. The research question is clinically relevant, since reverse TSR is increasingly used in patients with intact rotator cuff. The justification for conducting this large NHS-funded trial at this point in time could be presented more clearly.

2. There is no clear definition of minimum surgeon experience. Without such a threshold, bias due to learning curve effects is likely.

3. Cross-over risk: intraoperative conversion from aTSR to rTSR is expected. The handling of this issue in the analysis should be described in more detail.

4. Sample size: the calculation assumes an 8-point difference in SPADI. It is unclear if this difference corresponds to the established MCID. A clearer link to clinical relevance is required.

5. Statistical analysis: the description is brief. A detailed Statistical Analysis Plan (SAP) should be uploaded as an appendix.

6. Follow-up: the main endpoint is at 24 months. Since revision rates often occur later, a plan for longer-term follow-up should be discussed.

7. SPIRIT/CONSORT protocol checklist is broadly followed, but some elements (handling of missing data, subgroup analyses) need more detail.

The study is valuable, but the issues listed above should be addressed.

VERSION 1 - AUTHOR RESPONSE

Reviewer: 1
Dr. Antti Launonen, Tampere University Hospital
Comments to the Author:

Anatomic versus reverse total shoulder replacement for patients with osteoarthritis and intact rotator cuff: the RAPSODI-UK randomised controlled trial protocol

Thank you for the opportunity to review this necessary RCT. I think we need to investigate the limits to fill our knowledge gaps and push the boundaries. This trial is one of those important ones that help us choose the best treatment methods in the

future.

Even meticulously written, I would like to give some perspective and suggestions to consider in (hopefully) improving the trial. I acknowledge the sister trial in Australia has already started, and this one should be similar in main points.

The foremost concern I think should be addressed, either by discussing or creating an analysis around it, is the rTSA patients who are in high demand. E.g. construction workers or very fit patients going to the gym (lifting 100+kg), near 60 years old. At least in the North, we give rTSA patients an absolute lifting limit of max 10kg after the surgery. Naturally, that is something out of the blue – not investigated in any way, just as a mechanical consideration. However, that should be addressed in some way within the protocol, especially when the patients are also blinded to the allocation. For me, the optimal solution would be to analyse the result, if occupation, patient fitness or hobbies have affected loosening or revision rate. Or then just have a cutoff and exclude such patients.

Response: Exercise and heavy work may indeed have an effect on outcome and survival but is not evidence based as far as we are aware. Recruitment to the study is almost complete and we have not collected baseline data to specifically explore this suggestion. As the reviewer notes, the most likely effect will be on long-term survival which is outside the range of this protocol. With a two-year follow-up, we would expect low loosening or revision rates to be insufficient for a meaningful analyses.

The introduction is a bit lengthy. I think PPI could be placed in another part of the manuscript, and some elements of the introduction could be used in a discussion, which is now lacking. In the discussion, there could be more considerations of different patient populations and safety.

Response: The PPI section has been moved to the Discussion on page 25 and we have edited the introduction to shorten it where we can.

In cost-effectiveness analysis, indirect costs could be included, if possible. As mentioned earlier, the patients at work may be most affected by different types of prostheses or may not be able to continue in their work. This would be an essential aspect as well.

Response: Thank you for your helpful suggestion. Our protocol includes a planned secondary analysis from a wider societal perspective to account for indirect costs, in addition to the primary analysis from the NHS/PSS perspective. We have now expanded on the details of this in the 'Cost-effectiveness' section on p22 as suggested.

There are some points in the inclusion criteria;

As there are no formal indications for the OA surgery in the shoulder, I think the degree of OA should be recorded and analysed between the two groups to maintain similar conditions.

Response: We have not recorded the degree of OA at baseline, although we do record (1) whether there is any superior wear/superior deficit; (2) classification of the glenoid using the modified Walch system; and (3) grading of fatty infiltration. In a randomised trial with over 400 participants, the two groups should be balanced in degree of OA.

US is very low in specificity to detect the cuff rupture, especially in patients with advanced OA. I am not sure if it's an eligible way to inspect the cuff integrity within the trial.

Response: There have been surveys carried out establishing many UK surgeons use US as their modality of choice for establishing cuff integrity. We agree that MRI is a more accurate modality but allowing surgeons to utilise US reflects current UK NHS practice and the pragmatic design of the trial.

"Minimal glenoid erosion" is very shady and may be different from clinician to clinician even in a pragmatic setting. I would also add an analysis of the degree, with e.g., Walch classification.

Response: We are recording the Walch classification to be reported on at baseline.

P10, l 20 Not absolutely sure, but according to GCP, a signed consent should be sought in all times? Patients should be present in the treatment negotiation, so it should be possible?
Response: If this comment is referring to remote consent (e.g. over the phone), then the patient confirms their willingness to participate and when next come in hospital, they physically sign the Verbal Consent Form as approved by REC/HRA.

What comes to outcomes, did you consider WOOS? There are some clinical registry data available around WOOS in the NARA registry, so the trial outcomes could be mirrored with registry data.

Response: We explain in the "Outcomes" section why we selected the outcomes included in the trial. We are collecting the Oxford Shoulder Score from trial participants to enable comparison of the trial population with the National Joint Registry dataset in the UK. We did initially consider the Constant-Murley Score but quickly moved to the SPADI, as the Constant-Murley was less well validated. In

addition, the WOOS is used less in Europe and the UK, and as such, clinicians and researchers would not be familiar with using it.

The primary outcome time is from randomization. Why not from the treatment? I think the waiting list from randomization to surgery may be lengthy in some instances, so the actual outcome time is less than two years.

Response: We follow up participants from the point of randomisation as that preserves the baseline balance between groups achieved by randomisation and adheres to the intention-to-treat principle. Follow-up from date of surgery can introduce non-comparability and lead to biased results. Randomisation is undertaken two weeks prior to when surgery is planned to minimise the effect on length of follow-up.

I am not familiar with Teleform software, but from the protocol, I understand you are managing papers. Why not use something like Redcap or a similar tool? I have had a hard time collecting all the paperwork of 250 patients, and it is very time-consuming, and human error possibilities are very high, resulting in the loss of some patient data. After going all electronic, the data is secured once and for all. No missing data, and you can see the progression of the trial by checking the main view from the program desktop.

Response: Yes, we are collecting data postally using paper CRFs. We have robust systems to check for missing data and verification of data that are recorded in our clinical trials unit Standard Operating Procedures. Our experience in more elderly populations is that paper CRFs are well completed. Our experience from other trials using REDCap is that electronic data capture by no means ensures better response rates and/or less missing data and has its own limitations.

The statistical methods section is quite brief. Will you create a statistical analysis plan? Please mention it if that will be done.

Response: Yes, we will develop a Statistical Analysis Plan (SAP) and now reference that in the "Statistical Methods" section on p19.

Reviewer: 2
Dr. Nikolai Ramadanov, University Hospital Brandenburg/Havel Brandenburg Medical School, Brandenburg Medical School Theodor Fontane
Comments to the Author:

1. The research question is clinically relevant, since reverse TSR is increasingly used in patients with intact rotator cuff. The justification for conducting this large NHS-funded trial at this point in time could be presented more clearly.

Response: The Introduction summarises the lack of evidence to inform current practice. This includes the evidence gap identified in a Cochrane review, referring to data on differential revision rates in the two procedures from the from the National Joint Registry and the identification of the research question as a priority by the James Lind Alliance and NICE. We have edited the introduction to make this clearer.

2. There is no clear definition of minimum surgeon experience. Without such a threshold, bias due to learning curve effects is likely.

Response: To reflect the pragmatic design, a required level of experience of the operating surgeon is not defined, although all surgeons performing TSR for patients within the trial will be required to be familiar with the techniques and equipment that they are using. Data will be collected on the grade and experience of the primary and secondary surgeons through the NJR minimum data set.

3. Cross-over risk: intraoperative conversion from aTSR to rTSR is expected. The handling of this issue in the analysis should be described in more detail.

Response: Thank you, the impact of crossover will be considered in an additional analysis with the use of complier average causal effect modelling. Further detail about this analysis has now been included in the protocol on p21.

4. Sample size: the calculation assumes an 8-point difference in SPADI. It is unclear if this difference corresponds to the established MCID. A clearer link to clinical relevance is required.

Response: As our trial compares two active treatments, we expected a relatively small difference in SPADI score between groups at 24 months. Based on a previously published study, our target difference between the rTSR and aTSR groups is 8 points (the smallest published, validated difference classed as clinically important (Paul et al., 2004)). We have amended the text on p17 to better reflect this point.

5. Statistical analysis: the description is brief. A detailed Statistical Analysis Plan (SAP) should be uploaded as an appendix.

Response: We have added text clarifying that the statistical analyses will be detailed fully in a SAP agreed by the independent Data Monitoring and Ethics Committee (DMEC) prior to the end of data collection. This will be provided as Supplementary Material when we submit the results of the study for publication as per usual practice.

6. Follow-up: the main endpoint is at 24 months. Since revision rates often occur later, a plan for longer-term follow-up should be discussed.

Response: The section on "National Joint Registry Linkage" refers to our aim to collect longer term implant survival outcomes from the NJR and participant questionnaire follow-up data subject to the availability of further funding.

7. SPIRIT/CONSORT protocol checklist is broadly followed, but some elements (handling of missing data, subgroup analyses) need more detail.

Response: Further detail about the proposed statistical analysis has now been included in the protocol on p21.

The study is valuable, but the issues listed above should be addressed.

VERSION 2 - REVIEW

**Reviewer**	**1**
**Name**	**Launonen, Antti**
**Affiliation**	**Tampere University Hospital**
**Date**	**24-Oct-2025**
**COI**	

Brilliant trial!
